# Photosynthesis at the forefront of a sustainable life

**DOI:** 10.3389/fchem.2014.00036

**Published:** 2014-06-12

**Authors:** Paul J. D. Janssen, Maya D. Lambreva, Nicolas Plumeré, Cecilia Bartolucci, Amina Antonacci, Katia Buonasera, Raoul N. Frese, Viviana Scognamiglio, Giuseppina Rea

**Affiliations:** ^1^Molecular and Cellular Biology - Unit of Microbiology, Institute for Environment, Health and Safety, Belgian Nuclear Research Centre SCK•CENMol, Belgium; ^2^Institute of Crystallography, National Research Council of ItalyRome, Italy; ^3^Center for Electrochemical Sciences-CES, Ruhr-Universität BochumBochum, Germany; ^4^Division of Physics and Astronomy, Department of Biophysics, VU University AmsterdamAmsterdam, Netherlands

**Keywords:** photosynthesis, photosynthetic yield improvement, bioremediation, biofuels, biosensors, artificial photosynthesis, sustainability, space agriculture

## Abstract

The development of a sustainable bio-based economy has drawn much attention in recent years, and research to find smart solutions to the many inherent challenges has intensified. In nature, perhaps the best example of an authentic sustainable system is oxygenic photosynthesis. The biochemistry of this intricate process is empowered by solar radiation influx and performed by hierarchically organized complexes composed by photoreceptors, inorganic catalysts, and enzymes which define specific niches for optimizing light-to-energy conversion. The success of this process relies on its capability to exploit the almost inexhaustible reservoirs of sunlight, water, and carbon dioxide to transform photonic energy into chemical energy such as stored in adenosine triphosphate. Oxygenic photosynthesis is responsible for most of the oxygen, fossil fuels, and biomass on our planet. So, even after a few billion years of evolution, this process unceasingly supports life on earth, and probably soon also in outer-space, and inspires the development of enabling technologies for a sustainable global economy and ecosystem. The following review covers some of the major milestones reached in photosynthesis research, each reflecting lasting routes of innovation in agriculture, environmental protection, and clean energy production.

## Introduction

Sustainability has been described by the European Union as follows: “Strictly speaking sustainability implies the use of resources at rates that do not exceed the capacity of the Earth to replace them.” (http://ec.europa.eu/environment/eussd/food.htm). Sustainability however does not come about by its own but requires dynamic and responsible actions to create and maintain a balance between society, environment, and economy. In the next 10–20 years, a steady increase of the global population, continuous competition for land, water, and energy, and the worsening effects brought by climate change will be the three foremost science policy-determining factors. Innovations in agriculture no doubt will have a great impact on sustainability since they would address the usage and purification of water, the availability of good-quality soil, the supply of energy including “green” energy, the importance of biodiversity, and above all, the command and safety of food and feed. It is mandatory however that this is done in an interdisciplinary way following a holistic approach.

Photosynthesis is one of the most efficiently cycled and sustainable processes we know in Nature. This deceivingly simple process forms the basis for all the energy sources essential to life, from the intake of food to the burning of fossil fuels, and more recently, for the industrial production of value-added chemicals or bio-energy. Green plants, algae and cyanobacteria are able to oxidise water for photosynthesis and hence are oxygenic, in contrast to other phototrophs that use different electron donors, such as hydrogen sulfide. The splitting of water (H_2_O) giving off oxygen gas (O_2_) is a complex event, requiring the absorption of solar energy by a set of aligned chlorophyll (Chl) pigments that as a result release electrons to convert CO_2_ to carbohydrates, a reaction known as carbon fixation. The full process can be summarised in a single equation:

$${\text{H}}_{2}\text{O}+{\text{CO}}_{2}+\text{light}\to \text{C}({\text{H}}_{2}\text{O})+{\text{O}}_{2}$$

Oxygenic photosynthesis evolved approximately 2.5 billion years ago, the core reactions, light harvesting, charge separation, water splitting, and energy storage remaining similar across species. However, natural optimization of the mechanism through a series of fine physical and biochemical modifications allowed adaptation of the process to specific ecological niches. For instance, the evolution of C3 into C4 pathways enabled plants to attain, under certain conditions, an efficiency increase of their photosynthetic processes of up to 50%.

This review reports on research inspired by photosynthesis, addressing global, environmental and societal issues related to crops improvement, eco-system homeostasis maintenance and clean energy production, with the aim to identify opportunities and challenges for sustainable innovation and development.

## Photosynthesis at the forefront of a secure food supply

The global demand of nutrition largely depends on photosynthesis efficiency. Presently achieved crop yields however lay far below the projected needs required to meet the predicted population growth, threatening global food security (Fedoroff et al., 2010; Long, 2014). Current photosynthesis research is much inspired by the call for a sustainable agriculture and the tuning of food, feed, and energy production in respect to each other (Nair, 2014). The main challenge however lays in increasing crop yields without encumbering land and water resources nor by burdening the environment with an excess of herbicides or nitrogen-rich manure. Analyses of current knowledge and outputs of biochemical and microclimatic photosynthetic models indicate that, by exploiting variations of existing germplasms and by engineering photosynthesis (Zhu et al., 2010a; Reynolds et al., 2011; Gu et al., 2014), sustainable yield increases of crops could be achieved. The present section encompasses recent advances to increase the radiation-use efficiency (RUE) of crops, hence production yields, by overcoming natural photosynthetic limits and improving light perception.

### Overcoming the main limits of C3 photosynthesis

Plant growth and biomass production rely on carbohydrate synthesis within the Calvin-Benson cycle, which is initiated by the incorporation of CO_2_ into ribulose 1,5-bisphosphate (RuBP) by ribulose 1,5-bisphosphate carboxylase/oxygenase (RuBisCO). Eukaryotic RuBisCO comprises eight large (LS, chloroplast-encoded; containing the active sites) and eight small (SS, nuclear-encoded) subunits, organized in L_8_S_8_ macromolecules (Whitney et al., 2011a). RuBisCO catalysis requires pre-activation via an ancillary enzyme, RuBisCO activase (RA). It involves a complex 5-step reaction, and is further complicated by electrostatic similarity between CO_2_ and O_2_, as well as regular enzyme inhibition and -reactivation (Andersson and Backlund, 2008). RuBisCO promptly reacts with O_2_, losing approximately 25% of previously fixed CO_2_ through the photorespiratory pathway. RuBisCO's dual function and its low carboxylation rate (*V*_*c*_) compromise its efficiency and are among the main factors determining the low RUE of C3 plants (Zhu et al., 2010b). Manipulation of RuBisCO's catalytic traits, modulation of its inhibition, or RA optimization were recognized as main targets in the struggle for the increase of crops yields (Raines, 2011; Whitney et al., 2011a; Parry et al., 2013; Mueller-Cajar et al., 2014). In addition, improving C3 RuBisCO catalytic turnover should diminish the required RuBisCO amount (normally representing approximately 50 and 25% of leaf protein and nitrogen content, respectively) increasing nitrogen use efficiency of crops.

Comparative analyses of natural RuBisCO variants demonstrated that an increase in the carboxylation rate is generally obtained at the expense of CO_2_ affinity (*K*_*m*_) and/or specificity (*S*_*C/O*_). The high *V*_*c*_ of C4 type RuBisCO, coupled to low CO_2_ specificity/affinity, requires restriction of the enzyme's access to atmospheric oxygen for best results. A more intriguing target is a variant RuBisCO which evolved in some red algae and possesses high *S*_*C/O*_, keeping *V*_*c*_ values similar to those in the C3 plants (Whitney et al., 2011a). Another promising approach is a detailed structural, biogenetic and catalytic characterization of RuBisCO forms developed under challenging growth conditions (Miller et al., 2013). The production of chimeric L_8_S_8_ complexes or manipulation of LS and SS is hindered by the complex RuBisCO biogenesis and spatial separation of the LS- and SS-encoding genes in the chloroplast and nucleus, respectively (Whitney et al., 2011a; Parry et al., 2013). However, a better understanding of RuBisCO folding and assembly (Liu et al., 2010; Kolesinski et al., 2013) has enabled the engineering of LS peptides with improved catalytic properties in *Chlamydomonas reinhardtii* (Zhu et al., 2010a; Maliga and Bock, 2011) and tobacco plants (Whitney et al., 2009, 2011b) resulting in increased photosynthetic rates and higher biomass. Furthermore, RuBisCO-mediated carbon fixation with C4-like catalysis (high *V*_*c*_ and high *K*_*m*_) was realized in rice by introducing SS from sorghum and eliciting functional chimeric L_8_S_8_ complexes (Genkov et al., 2010; Ishikawa et al., 2011).

Due to the natural behavior of RuBisCO enzyme to form stable intermediate complexes with sugar phosphates, its *V*_*c*_ declines with time and repeated interventions of RA are required to prevent the so-called enzyme fallover (Mueller-Cajar et al., 2014). Attempts have been made to reduce RuBisCO fallover by lowering its sensitivity to the specific by-products or by accelerating the transformation of RuBisCO inhibitors into less active metabolites (Parry et al., 2013). Phosphate-induced impediment of RuBisCO heightens with the increase in temperature and highlights the main flaw of RA: its low thermostability. It was demonstrated that expanding the temperature range of RA stability (via overexpression of RA from warm- into cool-season species) or improving RA thermostability (via replacing the endogenous RA by a more thermostable enzyme) can give cause to increased photosynthetic performances and yields under moderate heat stress (Kumar et al., 2009; Carmo-Silva and Salvucci, 2012).

Metabolic control analyses indicated that the RuBisCO carboxylation reaction is the controlling step in the efficacy of the Calvin-Benson cycle, particularly under high light, high temperature and low CO_2_ conditions (Zhu et al., 2010b; Raines, 2011). Investigations using antisense RNA silencing to reduce RuBisCO protein levels revealed additional enzymes able to control the C3-cycle efficiency by modulating the RuBP regeneration rate (Raines, 2011). However, the mechanisms that regulate carboxylation-cycle reactions must be fully understood in order to introduce appropriate modifications. It was shown that the increase in photosynthetic rate and concomitant gain in biomass owing to the overexpression of sedoheptulose-1,7-bisphosphatase in fact is highly species- and growth dependent and that the overexpression of transketolase actually affects plant growth negatively, probably due to changes in the C3-cycle carbon exchange balance (Raines, 2011).

**Figure 1:**
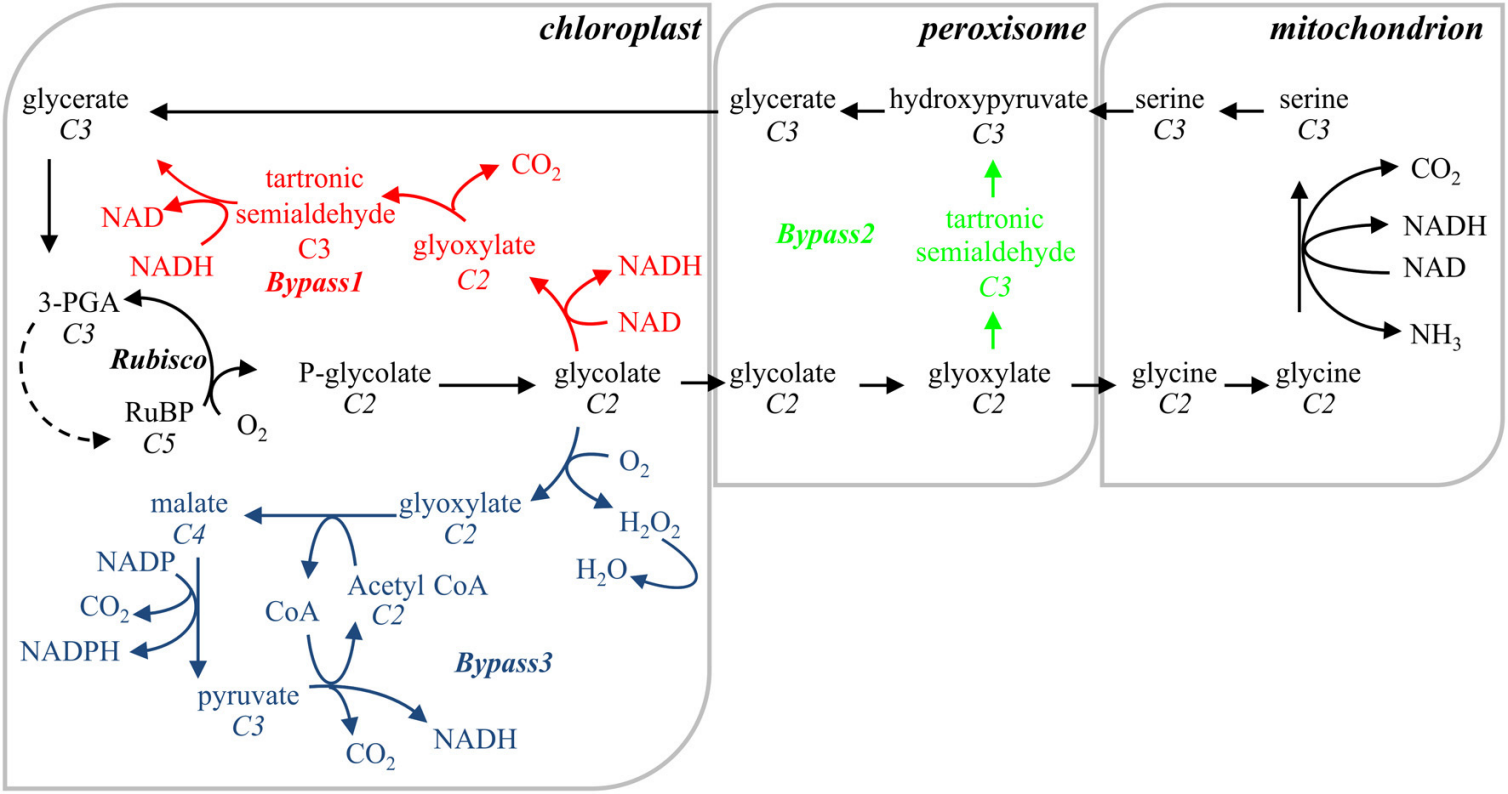
**Schematic representation of the photorespiratory pathway (in black) and the three circumvent pathways designed to overcome the photorespiratory losses**. The reactions of bypass 1 (in red) are entirely realized into the chloroplast and comprise the transformation of glycolate to glycerate, introducing glycolate dehydrogenase, glycine decarboxylase and tartronate semialdehyde reductase similar to the *E. coli* glycolate catabolic pathway (Kebeish et al., 2007). Bypass 2 (in green) follows the *E. coli* glyoxylate catabolic pathway in the peroxisomes by introducing glycine decarboxylase and hydroxypyruvate isomerase (Carvalho et al., 2011). Bypass 3 (in blue) oxidizes glycolate to CO_2_ in the chloroplast, using exogenous (glycolate oxidase and catalase from the peroxisomes, and malate synthase from the glyoxysomes) and endogenous (malic enzyme and pyruvate dehydrogenase) enzymes (Maier et al., 2012). In all three bypasses release of ammonia in the mitochondrion is abolished; 75% of the glycolate redirected toward bypasses 1 and 2 is returned to the Calvin-Benson cycle as 3-PGA; bypasses 1 and 3 dislocate CO_2_ released from the mitochondrion to the chloroplast. Reactions stoichiometry is not taken into account; the numbers of carbon atoms of each metabolic compound are in italic; 3-PGA, 3-phosphoglycerate; RuBP, ribulose 1,5-bisphosphate.

Parallel to the efforts to engineer RuBisCO proteins with increased *V*_*c*_, extensive research focused on increasing C3-cycle productivity by bypassing the photorespiratory pathway. Photorespiration entails the sequential transformation of phosphoglycolate (PG), the RuBP oxygenation product, back into glycerate to join the Calvin-Benson cycle in the chloroplast via a series of peroxisome- and mitochondrion-located reactions (Peterhansel et al., 2010). The regeneration of oxygenated RuBP consumes reducing equivalents and energy, and is associated with the release of CO_2_ and ammonia derived from previously fixed carbon and nitrogen (Peterhansel et al., 2013). Three different approaches to circumvent this “wasteful” lane have been proposed and tested in plants with different successes (Figure 1): (i) catabolization of PG to RuBP and CO_2_ in chloroplasts (Kebeish et al., 2007) (Bypas 1), (ii) a similar reaction in peroxisomes (Carvalho et al., 2011) (Bypass 2), and (iii) oxidization of PG to CO_2_ and pyruvate in chloroplasts (Maier et al., 2012) (Bypass 3). Bypasses 1 and 3 proved to be functional in *Arabidopsis* plants, and resulted in enhanced photosynthesis and growth. Both pathways are energetically less costly than the photorespiratory route, possibly avoiding ammonia re-fixation and releasing CO_2_ in chloroplasts (Peterhansel et al., 2010; Maier et al., 2012; Peterhansel et al., 2013). The integration of these basic approaches into agriculturally significant crops is still a challenge and the translation of their benefits into crop yields needs to be determined.

Some green algae and cyanobacteria are useful as feedstock and are regarded as safe for human consumption. Such microalgae generally perform C3 photosynthesis and rely on bicarbonate and CO_2_ transport. A carbon concentration mechanism (CMM) in green algae consists of a spatial regulation of carbonic anhydrase activity and the low-CO_2_ induced formation of a starch sheet around the pyrenoid, a proteinaceous structure where most (>90%) of the RuBisCo becomes encapsulated. In cyanobacteria a similar CCM exists, but here bicarbonates are transferred from the cytoplasm to a separate compartment, the carboxysome, which is not permeable by oxygen. Herein, a carbonic anhydrase converts bicarbonate to CO_2_ used as enriched substrate by the carboxysome-enclosed RuBisCo. It has been recently suggested that C3 plant photosynthesis might be improved through the introduction of microalgal bicarbonate pumps (Price et al., 2013) or complete carboxysomes (Zarzycki et al., 2013) or pyrenoids (Meyer and Griffiths, 2013) into their chloroplasts but these potential routes have not yet been demonstrated.

### Engineering C4 photosynthesis in C3 crops

Today, although only 3% of all vascular plants exhibit C4-photosynthesis, they account for one-fifth of the global primary productivity owing to ample high-yield C4-grass-lands. In general, C4 plants (i.e., maize, sugar cane, sorghum) are more efficient at photosynthesis than C3 plants and fix carbon at higher rates, use less water per weight of biomass produced, and tolerate to a better extent water and high temperature stress. But, in terms of ground use and taking into account that only parts of plants are edible, C3 crops (e.g., potato, soybean) produce some of the highest edible calories and protein per square meter. C4 photosynthesis is characterized by a two-step carbon fixation for the establishment of a CO_2_-enriched RuBisCO environment and creates conditions in which photorespiration is negligible. The majority of C4 species have higher RUE and biomass production than the C3 types, particularly in warm habitats (Langdale, 2011). These benefits are especially evident in species which evolved CCM, spatially separating the carbon fixation and carboxylation between the chloroplasts of mesophyll cells (MCs) and bundle sheath cells (BSCs), also known as Kranz anatomy or the Kranz-type C4 cycle. In contrast to C3, both C4 cell types are rich in chloroplast: MC chloroplasts possess a granal-stromal lamellae organization and lack the enzymes involved in the Calvin-Benson and photorespiration pathways; BSCs show predominantly stromal lamellae and a typical C3 enzymatic profile. C4 metabolism invests in enlarged and photosynthetically active BSCs, a high number of veins, a heavy metabolite traffic, and the functional coordination between BSCs and MCs. The introduction of a C4 cycle into typical C3 species, such as rice and soybean, is predicted to increase crop yield, with concomitant improvement in water and nitrogen-use efficiency (Zhu et al., 2010b; Langdale, 2011; Wang et al., 2011). The difficult transfer of high-yield C4 metabolism in important C3 crops, such as rice, became one of the main goals of the international C4 Rice Consortium (http://c4rice.irri.org/) established less than 10 years ago and aiming to address the growing food demand (von Caemmerer et al., 2012). The production of rice with acquired functional C4-biochemistry requires modification and integration of numerous biochemical pathways and adequate adaptation of rice leaf anatomy. The main approaches undertaken for the realization of this ambitious project have been thoroughly described (Kajala et al., 2011; Langdale, 2011; Leegood, 2013; Karki et al., 2013) and include: (i) the integration in rice of genes typical for the C4 metabolism, reaching expression levels conferring high photosynthetic yields, such as the genes encoding phosphoenolpyruvate carboxylase in MCs and enzymes from Calvin-Benson cycle in BSCs, (ii) the down-regulation of endogenous rice genes, e.g., encoding MC enzymes of the Calvin-Benson cycle and photorespiration, (iii) the introduction of C4 cell-type specific gene expression and protein accumulation in rice, including identification of suitable regulatory elements to ensure the protein's compartmentalization between MCs and BSCs, and (iv) the identification of C4 transporters carrying out the metabolite transfer between the subcellular compartments and the introduction of the corresponding genes into the rice genome. Significant progress was made through the identification of gene promoters for compartmental gene expression (Wang et al., 2013a), gene cloning for the main enzymes of the C4 metabolic pathway from maize and their transformation into rice (Kajala et al., 2011), and the determination of candidate transporters of intermediate metabolites between MCs and BSCs (Karki et al., 2013).

Another important challenge is altering rice leaf anatomy and morphology in order to make it comparable with the Kranz-type biochemistry. The BSCs of rice, a typical C3 plant, contain too few chloroplasts to attain high-yield C4 photosynthesis (Karki et al., 2013). This hurdle can be bypassed by cell-type specific overexpression of genes implicated in chloroplast development, thylakoid stacking, and photosystems assembly such as the homologous gene pair of the *Golden2-like* (*GLK1, GLK2*) transcription factors (Langdale, 2011; Karki et al., 2013). The confirmation of a differential accumulation of GLKs in maize MCs and BSCs and their regulatory role in dimorphic chloroplast differentiation (Wang et al., 2013b) proved this approach to be correct. Even more encouraging was the successful introduction of Kranz anatomy trails in C3 leaves, which caused an increase in vein density and larger BSC cells in an oat-maize chromosome addition (OMA) line (i.e., *Avena sativa* plants containing a maize chromosome) (Tolley et al., 2012). A large interaction surface and closed contact between MC and BSC are mandatory requirements for fulfilling the needs of the heavily loaded intercellular metabolite exchange typical for the C4 cycle. Recently, the first gene (*scarecrow*) responsible for the C4 specific leaf patterning could be identified (Slewinski et al., 2012; Wang et al., 2013a) while a better knowledge of Kranz-type leaf structure development and the underlying evolutionary mechanisms will be instrumental to engineer high-yield photosynthesis in rice (Slewinski, 2013).

### Improving canopy photosynthetic performance

Improvements of the photosynthetic efficiency on chloroplast/leaf level can lead to an increase of crop yield if the engineered traits can be effectively introduced on plant canopy level. In this context, the main concern is the unequal light distribution within the plant canopy, resulting in an excessive light overload at canopy surface and severe light limitation within the lower canopy levels (Zhu et al., 2010b, 2012). Improving the efficiency in which crops are able to convert intercepted light into biomass requires the identification of an ideal canopy architecture (thus optimizing canopy performance) while keeping in mind the species- and habitat-specific requirements. A possible approach is the breeding or engineering of plants with more erected leaves and dwarf phenotypes causing a better canopy light distribution and conversion while reducing stem investment and lodging losses. Mathematical models can estimate quite accurately the canopy distribution of the light-limited and light-saturated photosynthesis in different species and environmental conditions, thus assisting the identification of improved crop specific architecture (Zhu et al., 2012; Song et al., 2013). To this end, considerable progress was made in unraveling the genetic basis of plant architecture (Jin et al., 2008; Wang and Li, 2008; Zheng and Liu, 2013).

Additional tactics to improve canopy RUE have also been explored. One of the most commonly used approaches is the reduction of leaf chlorophyll content, i.e., by light-harvesting antenna complex (LHC) size reduction, to effectively diminish excess light caught by upper-canopy leaves. The superfluous absorbed light causes a photoinhibitory inactivation of the photosynthetic reactions and is largely dissipated through non-photochemical quenching mechanism. In addition, this approach would cause an increase in light availability in the lower-canopy levels, relieving their light-limited photosynthesis (Zhu et al., 2010b; Ort et al., 2011). Furthermore, increased canopy light penetration could also positively alter the heat canopy balance by lowering the temperature in the upper portion and increasing it near to the soil, thus improving crop yield (Ort et al., 2011).

The adjustment of chlorophyll content and composition as well as truncation of light-capturing antenna has been successfully applied for an improvement of RUE and an increase in biomass yield for microalgal mass cultures. Among the different strategies to accomplish this, silencing or down-regulation of LHC encoding genes proved to be effective, leading to a higher cell culture density, less fluorescence quenching and a better photosynthetic quantum yield, while algal cultures were more tolerant to oversaturating light intensities (Kirst et al., 2012; Gimpel et al., 2013). In contrast, *Synechocystis* sp. PCC 6803 mutants with truncated phycobilisome antenna showed reduced growth rate and whole-culture biomass production (Page et al., 2012; Liberton et al., 2013). For crops, the benefits of LCH size reduction in terms of season-long carbon gains have yet to be rigorously tested (Ort et al., 2011). Other lines of interest are the discovery of the hitherto unknown chlorophyll *f* displaying a red-shifted absorption maximum (Chen et al., 2010) and the reconsideration of the photosynthetic potential of chlorophyll *d* (Mielke et al., 2013). These findings propounded the idea to improve plant RUE by extending the by plants usable spectrum of photosynthetically active radiation, or PAR (Chen and Blankenship, 2011; Blankenship and Chen, 2013). Besides the development of all these strategies to boost high photosynthetic yields, the possibility to improve photoprotection capacity in crops should be kept in mind as well (Murchie and Niyogi, 2011).

## Bioremediation and biomonitoring based on photosynthesizers

Synthetic fertilizers and agrochemicals play a major role to meet the ever increasing global demand for food. However, the excessive and improper use of these chemicals has turned agriculture into a major source for environmental pollution. A fine example is the use of nitrogen-rich fertilizers which, while allowing for a tremendous increase in crop productivity, resulted in nitrate and nitrite run-offs that have heavily disturbed many lake and river ecosystems. In addition, the ammonium required for fertilizer production is industrially made via the Haber process utilizing up to 5% of the global natural gas production. Thus, the current use of such fertilizers translates in massive CO_2_ emissions arising from the nitrogen extraction, the transport of raw materials and products, as well as the actual fertilizer production.

Adjusting fertilizer input to avoid excessive runoffs and preserve fossil gas resources have become priorities for modern farming practices. In particular, the development of field sensing methods, such as deployed for nitrate (Plumeré et al., 2012a,b; Plumeré, 2013), may allow farmers to better estimate the fertilizer needs of crops. In parallel, remediation of contaminated water, soil, and atmosphere has become exigent. Commonly used nitrate removal methods include chemical precipitation, ion exchange, electrodialysis, and reverse osmosis. While these strategies may be applied to clean up contaminated water, they are impractical for *in situ* use, come at an additional energy cost, and cause further CO_2_ emissions. Instead, solar light driven microbial processes are applicable *in situ* and carry a greater promise for environment-friendly bioremediation.

The general term bioremediation refers to a number of waste management techniques involving the use of plants or microbes (bacteria, yeasts, fungi, algae) to eliminate or reduce the concentration of pollutants from a contaminated site (reviewed by Bhatnagar and Kumari, 2013; Willscher et al., 2013). Lately, the term phytoremediation has been adopted for the specific use of plants, while the term phycoremediation is now specifically applied to the use of (green) algae or cyanobacteria. Phytoremediation may consist of one or more of six different phytotechnologies (phytotransformation, rhizofiltration, phytostabilization, phytovolatilization, evapotranspiration, and phytoextraction) depending on the used plant and the type and depth of contamination (Paz-Alberto and Sigua, 2013; Moosavi and Seghatoleslami, 2013). Phycoremediation equally benefits from the use of oxygenic photosynthesis but the use of microalgae and cyanobacteria offers some advantages including a higher biomass productivity, a much faster growth, an easier control of cellular response, the avoidance of arable land use, and the ability to extract micro- and macronutrients from wastewaters or industrial flue gasses (Anemaet et al., 2010; McGinn et al., 2011; Pittman, 2011). Plants, microalgae, and cyanobacteria have been studied extensively in recent years as effective accumulators, biosorbents, and degraders useful for the bioremediation of different kinds of organic and inorganic pollutants (Olguín, 2003; Gosh and Singh, 2005; Mehta and Gaur, 2005; Marmiroli et al., 2006; Lone et al., 2008; Chinnasamy et al., 2010; Kong et al., 2010; Wang et al., 2010; De Philippis et al., 2011; Moosavi and Seghatoleslami, 2013; Paz-Alberto and Sigua, 2013).

Coupling the growth of microalgae on wastewater with energy production has been proposed since the 60's (Oswald and Golueke, 1960; Benemann et al., 1977; Hoffmann, 1998; Mallick, 2002; Rawat et al., 2011; Olguín, 2012; Fahti et al., 2013) while the efficiency of sustainable low-cost wastewater treatment based on microalgae has been confirmed in the past decade (de-Bashan and Bashan, 2010). In spite of these promising developments, several challenges and limitations exist for microalgal biomass in terms of aeration and adequate quantities of light (taking into account self-shading and turbidity in ponds or reservoirs, but also photoinhibition), fluctuations in temperature and pH, harvesting and extraction costs, and down-stream processing (Scott et al., 2010; Hannon et al., 2011; McGinn et al., 2011; Rawat et al., 2012). Yield and cost analyses for algal systems versus traditional fuel crops indicate that algal systems are not yet cost-effective (van Bellen, 2009; Beal et al., 2012; Slade and Bauen, 2013) and life cycle assessments (LCAs) such as recently reported by Passell et al. (2013) using commercial data for algal production of biodiesel are clearly wanted (see also section Evaluation of Environmental and Social Impacts of Biomass Energy production). Nonetheless, microalgal, and cyanobacterial systems that combine bioremediation (i.e., waste water treatment) or CO_2_ mitigation with the production of potentially valuable biomass—whether biofuels or other added-value products—remain attractive novel routes. In particular when (bio)technological advances, including genetic modifications, could improve their cost-effectiveness and enhance their role in global endeavors for a sustainable life. Hence, in the next sections of this review we mainly focus on microalgal bioremediation and biofuels.

### Microalgae for bioremediation

#### Phosphorus and nitrogen removal

Nitrogen and phosphorus are serious pollutants accumulating in waters as a result of agricultural runoff. The major effect of releasing wastewater rich in organic and inorganic chemicals such as nitrates and phosphates is mainly eutrophication (Correll, 1998), with consequent hypoxia or anoxia of aquatic animals. Microalgae, in this context, can offer an attractive solution as they are able to grow in wastewater conditions by utilizing the abundant organic carbon and inorganic nitrogen and phosphorus (Pittman, 2011) thus acting as bioremediators against these elements.

***Phosphorus***. Phosphorus is an essential element for all life forms. Autotrophs can assimilate this mineral nutrient only as an orthophosphate, i.e., after hydrolysis of its organic forms by extracellular enzymes. The presence of this element in soils is often limited owing to the formation of insoluble complexes. In general, water-soluble phosphate used in fertilizer suffuses, while less than 20% is absorbed by plants (Vance et al., 2003). Phosphate enters ground water, streams and rivers, and moves out to sea and oceans where it is directly consumed by marine phytoplankton thus entering the food chain (Baturin, 2003). Since microalgae accumulate phosphorus as polyphosphate bodies stored inside the acidocalcisome organelle (Seufferheld and Curzi, 2010), these photosynthetic organisms can be doubly useful: as bioremediators, to remove the excess of phosphorus from waters, and as temporary storage, to capture this macronutrient and return it to the terrestrial environment in form of agricultural fertilizer (Sivakumar et al., 2012).

***Nitrogen***. Plants, microbes and algae absorb nitrogen from soil or water and store it as biomass. Over time, the biomass decomposes releasing nitrogen into the soil (e.g., as ammonia, urea) or into the atmosphere (e.g., as N_2_O), where it may be recycled or lost. Although N_2_O is not produced in significant amounts in the presence of nitrates, it is not known if other nitrogenous compounds found in wastewater, such as urea and ammonia, are converted to this greenhouse gas. Since microalgae have unique natural mechanisms for removing excess of nitrogen, phosphorus and CO_2_ from water sources, these organisms have been widely investigated for nitrogen removal. *C. vulgaris* was used for nitrogen and phosphorus removal from wastewater with an average removal efficiency of 72% for nitrogen and 28% for phosphorus (Aslan and Kapdan, 2006), while other microalgae widely used for nutrient removal were *Chlorella* (Lee and Lee, 2001), *Scenedesmus* (Martínez et al., 2000), and *Spirulina species* (Olguín et al., 2003), next to *Nannochloris* (Jimenez-Perez et al., 2004) and *Botryococcus braunii* (An et al., 2003).

#### CO_2_ capturing

Microalgae have the ability to fix CO_2_ (generally via the Benson–Calvin cycle) with an efficiency 10–50 times higher than that of terrestrial plants (Li et al., 2008). Through the photosynthetic process, microalgae can completely recycle CO_2_, producing the chemical energy necessary for the completion of their vital functions. For this reason, CO_2_ mitigation by microalgae is still considered the best strategy for an efficient removal of this greenhouse gas and to address global warming, especially when combined with algal biofuel production (Wang et al., 2008).

Light intensity, temperature, and CO_2_ concentration strongly affect CO_2_ fixation, in the way that increasing light intensity while maintaining moderate temperature and moderate solute concentration, increases both fixation and CO_2_ solubility in liquids (Atkinson and Mavituna, 1991). One of the best sources of highly enriched CO_2_ is flue gas containing 10–20% CO_2_ from burning fossil fuels (Ge et al., 2011), but algal species differ in their apparent ability to use CO_2_ effectively. High level of CO_2_ inhibit some species, while others can thrive on CO_2_ levels up to 20% (certain strains of *Chlorella, Scenedesmus*, and *Cyanidium* even grow in 40–100% CO_2_—reviewed by Salih, 2011). The efficient mass transfer of CO_2_ to cells in the aqueous environment of large-scale liquid culture systems is therefore challenging. Nonetheless, efficient capture of CO_2_, NO, and SO_2_ for algal biomass production by directly introducing flue gas into microalgal cultures have been reported (Chiu et al., 2011 and reviewed by Van Den Hende et al., 2012).

#### Heavy metals

Heavy metals are known to cause, in human beings, various physiological disorders to hepatic, renal, respiratory, and gastrointestinal systems. The toxicity of heavy metals depends on their concentration, bioavailability, and chemical forms, and the duration of exposure. The ever-increasing contamination of aquatic bodies and soils by heavy metals is an issue of serious concern and challenge world-wide. Bioremediation of heavy metal-contaminated water employing various microorganisms, including microalgae, has been recognized as a cheaper, more effective and an eco-friendly alternative to the conventional physico-chemical remediation methods. Considerable research effort has been therefore devoted to the development of algal biosorbents able to remediate these pollutants (Hazrat et al., 2013).

Biosorption of metal ions from aquatic complex matrices is based on the interaction of metal ions with the functional groups on the surface or within the cellular wall of the algae biomass. This phenomenon clearly depends on the typical binding profile of the biosorbent. Therefore, different algal species having different sizes, shapes, and cell wall compositions will have different metal binding efficiencies (Monteiro et al., 2011).

Current methods used to treat heavy metal wastewater include chemical precipitation, ion-exchange, adsorption, membrane filtration, coagulation-flocculation, flotation and electrochemical methods, even if only the first three techniques are the most frequently studied, as recently reviewed by Fu and Wang (2011) and Vandamme et al. (2013).

Cyanobacteria that produce extracellular polysaccharides (EPS) have been successfully applied for the removal of a wide range of metals from water, including Co, Cu, Cr, Pb, and Zn (reviewed by De Philippis et al., 2011) and extracted biomasses of cyanobacteria have been used to adsorb radionuclides of Cs, Sr, Ra, and Am (Pohl and Schimmack, 2006). An interesting application of green microalgae (*Hydrodictyon sp., Oedogonium sp., and Rhizoclonium sp*.) for the bioremediation of heavy metals such as As and Cd, is described by Saunders et al. (2012), who reported an alternative and practical approach to algal bioremediation of metals in which algae are cultured directly in the waste water stream. Other green algae, i.e. *Closterium moniliferum* and *Coccomyxa actinabiotis*, which show efficient and selective radionuclide sequestration and are extremely radioresistant, are prime candidates for in situ biodecontamination in the nuclear industry (Krejci et al., 2011; Rivasseau et al., 2013). In the aftermath of the Fukushima nuclear accident the search for additional cyanobacteria and algae, but also aquatic plants, that can be deployed to efficiently eliminate radionuclides from the environment, has intensified (Fukuda et al., 2014). Another interesting study describes a system dynamics approach to explore the efficacy of using mixed microalgae populations to treat leachate-hypersaline water (Richards and Mullins, 2013). This model evaluates the temporal evolution of metal removal and lipid production using four common marine microalgae species (*Nanochloropsis, Pavlova lutheri, Tetraselmis chuii* and *Chaetoceros muelleri*) and shows that after a ten-day period, the microalgae population is able to remove over 95% of the metals from the solution, paving the way for new strategies of waste stream management based on the use of microalgae-based bioremediation coupled with lipid-production.

#### Remediation of solid-waste and wastewaters

Microalgae are also efficient agents for the assimilation of organic matter from various contaminated media. Different photosynthetic organisms, often microalgae/bacteria consortia, have been successfully used in the remediation of solid-waste and wastewaters containing pesticides (González et al., 2012; Jin et al., 2012), phenols (Chiaiese et al., 2011; Maza-Márquez et al., 2014), aromatic hydrocarbons (Ibraheem, 2010; Ghasemi et al., 2011), textile dyes and detergents (Sing-Lai Lim et al., 2010; Singh and Patel, 2012), primarily due to their capacity to metabolize these compounds as nitrogen, phosphorus, carbon, and sulfur sources. For detailed description of the mechanisms involved in the bioremediation of each aforementioned class of pollutants see McGinn et al. (2011).

#### Photosynthesis-based biosensors

Real-time monitoring of crop growth parameters and environmental field conditions are mandatory for the development of tailored-made strategies aimed at minimizing resource inputs while maximizing output and yield. In this context, biosensor technology revealed a more suitable tool compared to analytical conventional methods requiring sample pre-treatments and complex instrumentation.

Photosynthetic microorganisms offer versatile solutions for the construction of smart and sensitive biosensors, enabling the detection of even minute amounts of pollutants. The functional principle of these sensors relies on the interaction of some classes of herbicides, pesticides, or heavy metals with a specific pocket of the photosynthetic reaction centers. This evokes physico-chemical changes that can be easily converted into measurable electrical signals. For instance, mercury with a 10^−14^–10^−6^ M concentration range in industrial and agricultural effluents could be detected using *Chlorella* whole cell based biosensors (Singh and Mittal, 2012). Researchers have also shown that His-tag-purified reaction centers of *Rhodobacter sphaeroides* attached to a gold electrode are particularly suitable for specific biosensing of herbicides, as photocurrent generation was inhibited in a concentration-dependent manner by the triazides atrazine and terbutryn with a Limit of Detection (LOD) of 50 and 8 nM, respectively, but not by nitrile or phenylurea herbicides, opening up suitable protein engineering approaches to develop more sensitive and more selective biosensing devices for the control of weeds (Swainsbury et al., 2014).

Wild-type and genetically engineered strains of the unicellular green alga *Chlamydomonas reinhardtii* were exploited to develop a set of portable and easy-to-use biosensors. These sensors, making use of photosynthetic biorecognition elements, enabled fast and low-cost pre-screening of triazines, diazines, and ureas in water samples (Buonasera et al., 2009; Scognamiglio et al., 2009). Protein engineering and synthesis of biomimetic peptides also allowed the design of *C. reinhardtii* mutants or novel photosynthetic binding domains with enhanced stability or tolerance to free-radicals-associated stress and heightened sensitivity for triazinic and ureic herbicides (with an LOD in the range of 10^−8^ M) (Rea et al., 2009; Lambreva et al., 2013; Scognamiglio et al., 2013).

## The use of “natural photosynthesis” in solar-energy-converting technology

### Biomass energy: a carbon neutral resource

The extensive global use of fossil fuels greatly increased the release of CO_2_ and other greenhouse gases into the atmosphere leading to important climate changes. There is a dire need for alternative energy sources (Frölicher and Joos, 2010; van Kooten, 2013) and hence biofuels produced from photosynthetic organisms or organic wastes offer the great opportunity to address the world's dependence on oil and to reduce CO_2_ emissions.

While the burning of fossil fuels increases the CO_2_ levels in the atmosphere by releasing carbon sequestered millions of years ago, the use of biomass maintains a closed carbon cycle returning carbon previously incorporated by growing plants to the atmosphere (Abbasi and Abbasi, 2010; Kopetz, 2013). Different biomass sources show large variations in terms of yield, quality, and cost. In the past, biomass from food crops, hydrocarbon-rich plants, waste reuse, or weed and wild plants were investigated for energy production and it was shown that the efficiency of mass-to-energy conversion is related to their biomass composition, i.e., the quantitative proportion between the three main organic constituents cellulose, hemicellulose, and lignin (Irmak et al., 2013; Zeng et al., 2014).

Recently a new energy production line from biomass derived from photosynthetic microorganisms (e.g., algae) has been added to the already known carbon neutral methodologies. While there are still challenges, the results obtained so far show the potential of this innovative approach (Ghasemi et al., 2012; Menetrez, 2012; Adenle et al., 2013).

### Biofuels generations

The history of biofuels starts with Louis Pasteur in the 19th century who in 1861 observed butanol production from anaerobic fermentation (later on picked up by Chaim Weizmann in 1913 studying acetone-butanol-ethanol fermentations in clostridia growing on a large range of biomass, most notably molasses). In the 1890s, Dr. Rudolf Diesel invented his revolutionary engine designed to run on a wide range of fuels, including vegetable oils, followed by Nicolaus Otto's pioneering spark-ignition engine designed to run on ethanol (i.e., derived from plant mass). In the early 20th century petroleum became widely available and the biofuel concept gained little attention except for brief interests during World War II and the 1970s oil crisis. During the last few decades, interest for alternative energy sources was rekindled and different biofuels were introduced on the markets (Kovarik, 2013).

Current biofuels are classified depending on the feedstock type used. The first generation of biofuels, of which ethanol and biodiesel are the main exponents, are essentially derived from food crops such as soybean, wheat, sugar-cane, and corn, using different procedures depending on the type of “green fuel” to be produced (Lee and Lavoie, 2013). The production of ethanol is generally obtained by fermentation of C_6_ sugars (e.g., glucose) through the action of yeasts, such as *Saccharomyces cerevisiae*, while biodiesel production requires a chemical process (Park et al., 2013). The treatment includes the extraction of the oil fraction from the edible biomass and its transformation in biodiesel through trans-esterification in the presence of methanol to obtain methyl esters (biodiesel), with glycerol as a by-product. Eventually the green fuel is recovered by repeated washings with water to remove glycerol and methanol (Naik et al., 2010; Lee and Lavoie, 2013).

Despite environmental benefits, the first generation of biofuels was accompanied by concerns about potential drawbacks generated by the competition with food and fiber products as well as by the competition for land and water (Ajanovic, 2011; Lee and Lavoie, 2013; Mohr and Raman, 2013), rising doubts about costs and sustainability and prompting research into the second generation of biofuels.

The second generation of biofuels exploits the potential of cheap, plentiful, non-edible biomass of lignocellulosic nature. This biomass is divided in three subcategories: homogeneous (e.g., wood chips), quasi-homogeneous (e.g., agricultural or forest residues) and non-homogeneous (e.g., municipal wastes) (Naik et al., 2010; Lee and Lavoie, 2013). The production is based on thermochemical or biochemical processes generating different end-products.

The thermochemical pathway transforms the whole biomass into three phase fractions by heat treatment in the presence of different oxygen concentrations: solid (biochar), liquid (bio-oil) and gaseous (syngas), their relative percentage being dependent on the different thermal conditions applied. At low temperatures (250–350°C) and in the absence of oxygen, torrefaction occurs and mainly biochar is obtained; at higher temperatures (550–750°C) and in the absence of oxygen, bio-oil is primarily produced through pyrolysis; at very high temperatures (750–1200°C) and in the presence of traces of oxygen, a gasification process occurs, producing mostly syngas. In theory any lignocellulosic biomass can be treated with any of the aforementioned thermo-chemical processes. In practice however technical and economic restrictions need to be considered on a case to case basis (Abbasi and Abbasi, 2010; Naik et al., 2010; Gallagher and Murphy, 2013; Lee and Lavoie, 2013).

The biochemical approach, exclusively applicable on the cellulosic fraction of the biomass, consists of hydrolysis, fermentation, and product separation. The complexity of this method is due to the resistant nature of cellulose (usually requiring a pre-treatment), the plethora of sugars released after breakage, and the need of specific microorganisms (including genetically engineered ones) to obtain an efficient fermentation (Abbasi and Abbasi, 2010; Lee and Lavoie, 2013).

When it became apparent that biofuels based on (micro)algal biomass could potentially provide much higher yields with lower resource inputs, efforts concentrated on the third biofuel generation. Current microalgae culture systems exist as expensive photo-bioreactors or in weather-dependent and contamination-prone outdoor systems (Chen et al., 2011; Halim et al., 2012; Makareviciene et al., 2013), but they do not need valuable farmland and their impact on fresh water resources are minimal if water is recycled or when waste waters or industrial effluents are used. Their potential is also given by an excellent photosynthetic performance, a good tolerance to hostile environmental conditions (Singh et al., 2011a,b; Larkum et al., 2012; Allen et al., 2013; Borowitzka and Moheimani, 2013), and a great variety in content and lipid profiles depending on the used species, growth conditions, and medium composition (Halim et al., 2012; Nascimento et al., 2013).

The conversion process from (micro)algal biomass to biofuel starts with harvesting and dehydration. Collection can be realized by centrifugation, filtration, or flocculation. The latter method, having the lowest energy cost, is usually achieved through the addition of polymers to the suspension (Vandamme et al., 2013). After removal of most of the liquid, a pre-treatment (cellular decomposition by high-pressure homogenization, total dehydration, and milling to fine powder) is required to optimize the material for lipids extraction (Halim et al., 2012; Pahl et al., 2013) carried out by using organic solvents or supercritical fluids (e.g., highly pressurized liquid carbon dioxide—see below). Eventually the mixture is filtered to remove cellular debris, while extraction solvents and residual water are eliminated by distillation, vacuum evaporation or solid- phase solvent adsorption. The final product consists of a lipid crude extract, usually containing polar and non-acylglycerol lipids. Before trans-esterification, the fraction of non-acylglycerol lipids, considered a contaminant in the biofuels production, is removed by liquid chromatography, acid precipitation and urea crystallization. During the trans-esterification, fatty acids are converted to alkyl esters in the presence of alcohols. The final mixture is purified to remove chemical contaminants and finally allowed to settle by gravity. Within the biphasic mixture, the top part consists of the biofuel, whereas the bottom part is glycerol. After decantation and several washings, the composition of biodiesel is analyzed by gas chromatography (Halim et al., 2012; Makareviciene et al., 2013).

The production of fuel starting from a genetically modified algae biomass, capable of going beyond the sustainable production of energy, is the goal of the next generation of biofuels. This could be achieved through new algae strains that are able to capture and store excess CO_2_ thus acting as a carbon negative rather than carbon neutral source (Lü et al., 2011; Liew et al., 2014).

Despite many advantages, there are still technological and economic limitations in the production chain of biofuels from algae. The main obstacles are in the selection of algal strains and in the development of an efficient lipid extraction process economically favorable for production at the industrial scale (Aransiola et al., 2014; Liew et al., 2014).

In particular, the ideal (micro)algal strain for biofuel production should have high lipid productivity, high CO_2_ fixation capacity, limited nutrient requirements, a fast growing cycle, high photosynthetic efficiency, outcompete contaminant strains in open pond production systems, be able to produce valuable co-products, and display self-harvesting characteristics (Brennan and Owende, 2013). Currently, there is no (micro)algal strain with all these requirements. Though genetic engineering-inspired modeling will be the tool for success, this remains limited to a few algal laboratory model strains, and additional genome sequencing of strains with appropriate features is required. Microalgae can potentially produce 100 times more oil per acre land as compared to any terrestrial oil-producing crop (www1.eere.energy.gov/biomass; Singh et al., 2011a) which remains, in spite of the fact that the current cost of microalgae per unit mass is higher (Greenwell et al., 2010), an attractive point in terms of lowering fossil fuel dependency.

### Biorefinery

The generation of a variety of bio-based products next to the production of energy would significantly maximize the value of the used feedstock (Azapagic, 2014). This is achieved taking advantage of various components present in the biomass as well as by adopting different technologies and processes. In particular, microalgae and cyanobacteria show potential since, in addition to biofuel precursors, carbohydrates and cellulose (ideal for the production of fine chemicals), they also produce pigments (e.g., phycocyanine, carotene, astaxanthin) and antioxidants useful in pharmaceutical and cosmetical applications, vitamins, and bioactive peptides (e.g., with antihypertensive, anticoagulant, antiviral or antimicrobial activities), and can be very rich in proteins making them suitable for the food and animal feed market (Vanthoor-Koopmans et al., 2012; Jarda et al., 2013; Uggetti et al., in press).

A current trend in the biorefinery is the use of biomass from bacteria and algae associated with wastewater treatment (Olguín, 2012; da Silva et al., 2013; Rawat et al., 2013). The dual purpose of this system makes it one of the most promising strategies in microalgae exploitation owing to the cost-effective and competitive production. Additional studies are required, since there are still limits in the development of an efficient procedure for the separation of the different fractions and for the preservation of the compounds of interest.

### Evaluation of environmental and social impacts of biomass energy production

Conversion, use and accessibility of energy are basic concepts of sustainability, which need to be coupled to acceptable social, economic, and environmental dimensions. In order to guarantee societal benefits of biofuels production, governments, researchers and companies will need to cooperate in carrying out assessments, mapping suitable areas, defining, and applying national, and international standards, as well as enhancing commercial-scale production (Singh and Olsen, 2012; Rathore et al., 2013).

Lately, a new tool for sustainability assessment of biofuels has been introduced: LCA, which helps policy makers in their choice of the most appropriate biofuels for specific purposes (Clay and Fong, 2013; Kendall and Yuan, 2013).

According to the US Environmental Protection Agency (EPA) a complete LCA process of biofuels includes the evaluation and analysis of every single step of production, from raw material to harvesting, processing, and transport, to their storage and distribution, and to their final use (http://www.epa.gov/nrmrl/std/lca/lca.html). The LCA approach has proven a valuable method in understanding the environmental impacts generated by various industrial products during their production, and is now the foremost accepted methodology for the assessment of environmental impacts (e.g., eutrophication, soil erosion, water run-off, loss of natural biota, and land resources) related to the introduction of new-technology fuels (Singh and Olsen, 2012; Ajayebi et al., 2013).

### Biofuels: current situation and new scientific research trends

The global scenario of “green fuel” production is still imprinted by the presence of first generation biofuels. Main producers of bioethanol are the United States and Brazil and soybean and sugarcane sucrose biomasses are the traditional feedstocks for biodiesel (Koçar and Civaş, 2013; Avinash et al., 2014). However, a trend toward large-scale production of biofuel exists. In Australia the current strategy is to increase the biofuel industry potential by exploiting marginal lands for exotic plant cultivation, while in India many venture assets presently concentrate on the use of invasive and non-edible plants for alternative energy production, even if the use of invasive plants for biofuel is not economical nor sustainable.

Concerning the production of third generation biofuels, strong support comes from scientific research in Europe, China, and the United States, with an emphasis on biomass yield improvements and the exploitation of genetic engineering tools. Several strategies are under investigation to overcome the limitations of large-scale production and to expand the market of this alternative source of energy. In particular, studies on microalgae and plants are aimed to control carbon portioning during photosynthesis within the cells (Liberton et al., 2013; Melis, 2013). This could result in higher yields and quality improvements of the final products. A recent example is the heterologous expression of the entire isoprene biosynthetic pathway in the cyanobacterium *Synechocystis* PCC 6803 (Bentley et al., 2014).

Another experimental approach aimed at increasing the biomass productivity concerns the RUE enhancement by modulating light capture at the antenna complex. This can be achieved either by optimizing the light distribution within the plant canopy, by extending the PAR spectrum, or by size reduction of the light harvesting antenna (see section Improving Canopy Photosynthetic Performance). Genetic engineering plays a key role in the manipulation of microorganisms, addressing the improvement of their growth rate, production and accumulation of their metabolites and precursors, and harnessing their defenses against microbial competitors, while simplifying harvesting methods (Gimpel et al., 2013; Shurin et al., 2013; Leite and Hallenbeck, 2014). Bacteria have been engineered to produce a wide range of chemical compounds closely related to fuel molecules, and synthetic biology approaches were applied for the production of advanced biofuel targets (Choi and Lee, 2013; Gronenberg et al., 2013; Wen et al., 2013). Molecular biology also enabled the production of specific yeasts and algae used in biofuel platforms. In particular, engineered yeast cells producing alcohols, sesquiterpenes, and fatty acid ethyl esters have been used for advanced biofuels with improved yield. Furthermore algae have been genetically manipulated to optimize their fatty acid biosynthesis, hence to increase oil content, or to optimize acid chain length for more stable algal biodiesel (Buijs et al., 2013; Gimpel et al., 2013). The CoA carboxylase enzyme, involved in the rate-limiting step in fatty acid biosynthesis, is one of the primary targets for the increase of algal oil yields (Mühlroth et al., 2013). Likewise, to control fatty acid chain length and hence biodiesel quality, thioesterases that terminate the chain elongation in fatty acid biosynthesis and functionally determine the identity of the end product are targeted as well (Blatti et al., 2013).

Research also focuses on the improvement of technologies concerning the collection and the conversion of algal biomass. An efficient harvesting method is a major challenge for the algal biofuel commercialization. Although sedimentation and flocculation seem to be the best low-cost methods they are not suited for all microalgae strains (Vandamme et al., 2013). New methodologies need to be developed and efforts are currently directed toward genetic modification to improve algae collection through the promotion of cell aggregation and the ability to flocculate more easily (Mendez et al., 2009; Scholz et al., 2011).

A recent challenge related to the conversion of algal biomass into biofuel is to achieve a single-step procedure. The development of a “supercritical approach” allowing the direct conversion of wet algae to crude biodiesel is under investigation (Patil et al., 2012; Reddy et al., 2014). It has been shown that supercritical carbon dioxide extracts lipids from algae with more efficiency and higher selectivity than traditional solvent separation methods, while extract purity and the final product concentration remain high. A further improvement would be combining the process of supercritical CO_2_ lipid extraction with the use of a suitable solid catalyst to allow extraction and conversion at a single production site (Soh et al., 2014).

The hydrothermal liquefaction for biomass conversion is also an appealing procedure and is particularly suitable for the direct treatment of wet feedstocks (Kruse et al., 2013; Chen et al., 2014; Cheng et al., 2014; Li et al., 2014). This technology allows simultaneous production of value-added compounds and bio-oil from algal biomass while a one-step process for direct liquefaction and conversion of wet algal biomass under supercritical methanol conditions should be possible (Patil et al., 2011).

### Bio-based polymers

Plastics play a major role in a sustainable development. Being both affordable and highly versatile, they have become essential to meet necessities in sectors such as health, shelter, communication, transportation, and food and energy security. Considering the growing demand for polymer products it is necessary to identify those production procedures which provide the lowest environmental impact and carbon footprint. While the exploitation of fossil feedstock in the manufacturing of plastics represents a sustainable and effective use of oil and gas, the most successful approach to bio-based polymers is to produce monomers (having at least two reactive groups or one C=C bound) from bio-waste and from “renewable oil” obtained from biorefineries and through plastic degradation. This, in combination with a melt- and gas-phase polymerization process, provides renewable and bio-degradable plastics without impairing their intrinsic characteristics (Mülhaupt, 2013). Examples of bio-based, renewable monomers are ethylene (for poly vinyl chloride or polyethylene glycol) e.g., from bioethanol, diamines (for polyamides and isocyanates) e.g., from amino acids and sugars, phenols (for polycarbonate or polyepoxides) e.g., from lignin, and polyols (for polyurethanes) e.g., from carbohydrates.

## Solar-energy-converting technology mimicking natural photosynthesis

The energy efficiency of natural photosynthesis, defined as the ratio of energy content of the annually harvested biomass versus the annual solar radiation over the same area, rarely reaches 1%, with a theoretical maximum for algal biofuels of 4.5% (Blankenship et al., 2011; Barber and Tran, 2013; Frischmann et al., 2013). As a consequence, biomass-based energy production could provide a limited contribution to our future energy demand. However, the high efficiency of light-harvesting (quantum efficiencies >90%; defined as the probability that an excitation leads to charge separation; Şener et al., 2011) and light-driven water oxidation (>80% in low light conditions; Ananyev and Dismukes, 2005) inspire the development of innovative solar-energy-converting technologies.

### Photovoltaic cells

Photosynthesis consists of a subset of energy conversion processes which can be mimicked in solar energy harvesting devices (Andreiadis et al., 2011). At the most basic level this implies the conversion of sunlight into charge-separated states by which an electron is freed from a light-absorbing semiconductor material (often silicon doped with impurities). The empty space left behind at the site of the electron-emitting atom is often referred to as a “hole.” Photo-generated electrons are conducted toward an acceptor material while holes—which behave as mobile positively charged particles—diffuse into the opposite direction. This separation of hole-electron pairs (called “excitons”) into free electrons and holes is expressed as the photovoltage and is the source of usable electrical energy.

Thus, photovoltaic devices generate electrical energy under illumination by exploiting semiconductor material or molecular photosensitizer for light-induced charge separation. While photovoltaic technology is already implemented at a global scale, the search for cheaper and less energy demanding materials for device fabrication is ongoing. One class of alternative materials for light induced charge separation includes the components of natural photosynthesis, the photosystem protein complexes. Their extreme quantum efficiency and large natural abundances makes them highly suitable for biohybrid photovoltaic devices (Barber and Tran, 2013; Frischmann et al., 2013).

The major consideration is how to avoid charge recombination when photosynthetic biomolecules are integrated in photovoltaic devices since the charge carrier produced should not be quenched at the photoelectrode it originates from but readily transferred to the counter electrode, preferably close to the circuit for effective power generation. A number of complete photovoltaic cells based on photosystem 1 (PS1) and/or photosystem 2 (PS2) are described below.

The first generation of biophotovoltaic cells based on isolated photosynthetic proteins incorporated semi-conducting electron transport layers to interface the biomolecules with the electrodes (Das et al., 2004). A later concept followed dye sensitized solar cell (DSSC) technology. In DSSC, the intrinsic properties of semiconductor materials with high overpotential for specific electron transfer processes are exploited to limit charge recombination processes. The same principles were applied for the construction of a PS1-semiconductor hybrid photoanode (Mershin et al., 2012). The semiconductor properties coupled to the high surface area electrode yielded the highest energy conversion efficiency reported to date for a biophotovoltaic device (0.08%). Still, the performance of the hybrid system remained well below that of state-of-the-art photovoltaic devices. Moreover, biophotovoltaic concepts much rely on the properties of the semiconductor material. The energy-costly fabrication and the light-induced charge separation properties of the semiconductor may thwart the advantage of implementing the biological component. Thus, for photosystem-based hybrids to make any significant impact in sustainability schemes, suitably conductive and cheap electrode materials must be found.

**Figure 2:**
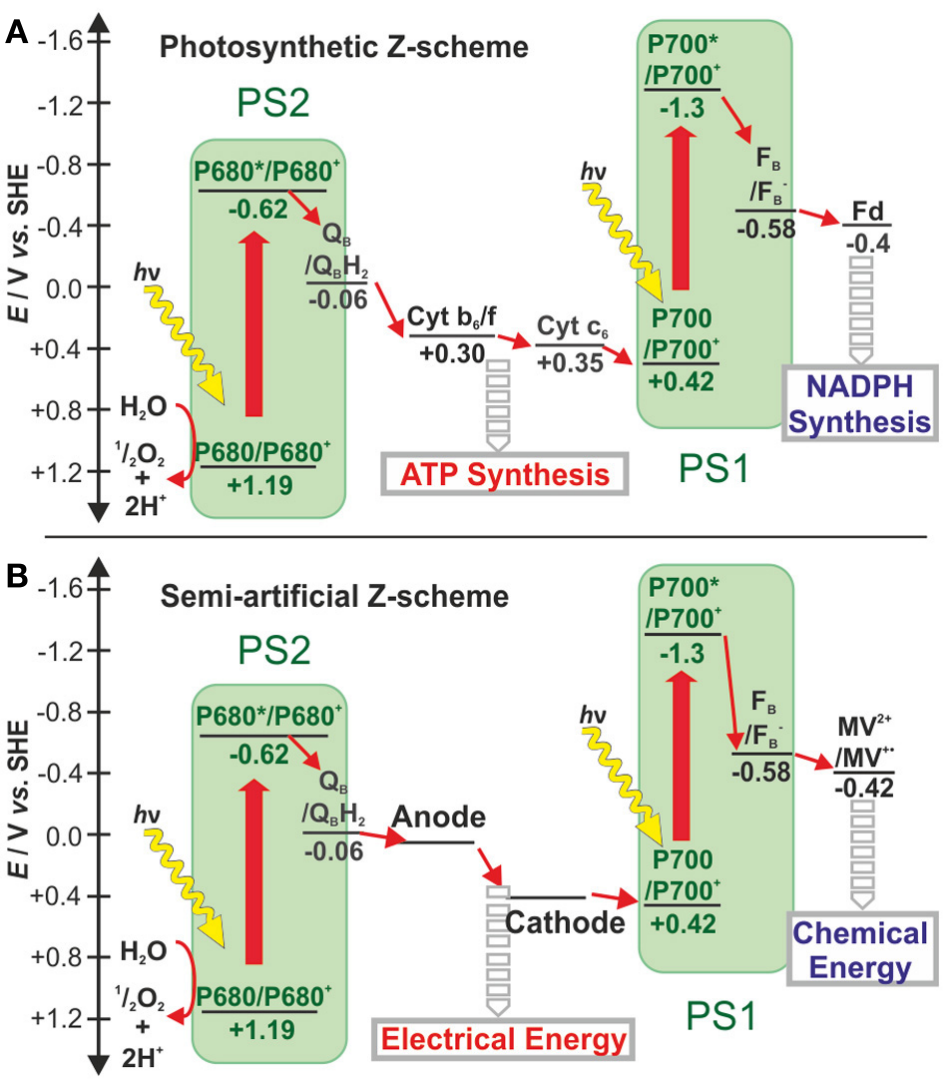
**Schematic comparison of electron transfer chains in (A) natural photosynthesis and (B) semi-artificial photosynthesys diplaying the Z-scheme for the conversion of solar light to electrical and chemical energy - adapted from (Kothe et al., 2013a,b)**. Electron transfer steps are shown as small red arrows while light-induced charge separation steps are depicted as large red arrows. In natural photosynthesis, the electron transfer from Q_B_ in PS2 to P700 in PS1 creates a chemiosmotic potential further exploited for ATP synthesis. The high-energy electrons exiting PS1 are transferred via Fd to FNR for NAPD^+^ reduction to NADPH. In the semi-artificial Z-scheme the electron transfer chain between PS2 and PS1 is shortcut by electrodes to recover the energy as electricity. In addition, PS1 transfers its electrons to methyl viologen, an artificial charge carrier. The latter is envisioned to mediate these electrons to catalysts such as hydrogenases for chemical energy production (Haehnel and Hochheimer, 1979). Cyt *c*_6_: cytochrome *c*_6_, C*b*_6_*f*:cytochrome *b*_6_*f* complex, Fd, ferredoxin; FNR, ferredoxin:NADP^+^ oxido reductase; MV^2+^, methyl viologen). Potentials are given in volt (V) versus the standard hydrogen electrode (SHE).

Yehezkeli et al. (2012) reported on a biophotovoltaic cell that fully excluded semiconductors but instead was based on a PS2 photoanode in combination with an oxygen reducing biocathode. Upon charge separation at the PS2 anode, water becomes oxidized and the produced oxygen diffuses to the cathode where it is reduced back to water via an enzyme-catalyzed process. The irreversible electrochemical properties of the charge carrier, oxygen, ensure that its reduction only takes place at the catalyst-modified biocathode. Charge recombination of oxygen at the PS2 photoanode is slow, and short-circuiting is therefore impeded. This concept was recently extended to the combination of a PS2 photoanode with a PS1-based oxygen-reducing photocathode (Kothe et al., 2013a,b; Hartmann et al., 2014). In this approach, oxygen is again exploited as the charge carrier. The additional charge separation step at PS1 allows to couple a catalyst for H_2_ evolution or other irreversible reductive processes. This provides the basis for the development of semi-artificial devices that fully mimic the two solar energy conversion steps in natural photosynthesis (Figure 2) (Kothe et al., 2013a,b; Hartmann et al., 2014).

The above illustrates how charge recombination can be avoided by exploiting irreversible charge carriers such as oxygen in the PS2-based devices. However, bringing PS1 in contact to a conductive electrode is more challenging since, in contrast to PS2, PS1 does not catalyze an irreversible chemical reaction but instead exchanges electrons with reversible redox partners. Hence, freely diffusing charge carriers will recombine with each other or with the electrode. The main strategies to minimize such recombination losses rely on (i) contacting both the electron-accepting and the electron-donating sides of PS1 with surface-confined electron relays or (ii) direct electron transfer between the two electrodes. Here, the challenge resides in the actual cell fabrication since PS1 needs to be inserted into a nanogap between the two electrodes. The underlying concept was shown at the single molecule level in a double junction involving direct electron transfer (Gerster et al., 2012). Although the electric current density and open-circuit voltage of such a bionanodevice opens up prospects for highly efficient biophotovoltaics, upscaling this concept to a large surface area may be difficult since defects in the nanogap would result in direct short-circuits between the two electrodes (Plumeré, 2012). A large-scale device that fully excludes the use of semiconductor material still needs to be demonstrated.

To date, efficiencies of devices that deploy biological components for light-induced charge separation have not yet exceeded 0.1%, which is well below those seen for standard semiconductor photovoltaics. A further improvement in performance requires alternative strategies to circumvent charge recombination, in particular for PS1-based devices (Kothe et al., 2014). One approach that has been overlooked so far is to mimic the role of NADPH in nature i.e., the role of O_2_ in the PS2-based system, namely to develop for PS1 an electrochemically irreversible charge carrier that requires a catalyst for electron transfer. Also, an enhanced loading of photosynthetic protein may eventually result in higher photocurrent densities. When the electronic communication between the photosynthetic protein and the electrode is based on a monolayer design, porous high-surface-area electrodes may be used. However, this would imply the use of transparent materials therefore rely on expensive semiconductors. Instead, the technological trend may move from monolayer layouts toward multilayers that are contacted via conducting polymeric matrices. In this case, transparency and electron transfer properties within the polymer-protein hybrid film must be further developed to realize highly efficient biophotovoltaic systems. Moreover, in the development of high performance biophotovoltaics, the stability under illumination is also a critical factor. In this regard, since PS1 is more stable as compared to PS2, the former may yet be better suited for the development of energy conversion devices.

### Fully-integrated artificial photosynthetic systems

The term artificial photosynthesis was coined for the first time to make a distinction between conventional silicon-based and novel dye-sensitized solar cells (DSSC) (Grätzel, 1991). In contrast to the direct electron transfers from the silicon atoms upon light absorption, the DSSC separates light absorption by “sensitized” metal-complexed or organic metal-free dye molecules which subsequently inject electrons into semiconducting material. This way, energy conversion efficiencies of up to 10% can be obtained (Nazeeruddin et al., 1993). Although the original DSSC necessitates a liquid electrolyte, affecting device stability, recent advances have shown the possibility for these solar cells to perform well as solid state devices using hybrid perovskite dyes (Burschka et al., 2013). Perovskite-based technologies and architectures for solar cells are constantly being improved and they show great promise for the realization of artificial photosynthesis (Zhou et al., 2013; Christians et al., 2014).

The success of the DSSC in terms of efficiency and cost effectiveness has been an important driver for artificial photosynthesis research targeting the more complex energy conversion processes of natural photosynthesis. A major target to mimic are the reactions that involve the splitting of water. While in natural photosynthesis the protons form a gradient across the cell membrane to drive ATP synthesis, energy conversion devices rely on the subsequent formation of molecular hydrogen as a combustible fuel. In the realm of semiconducting materials, water splitting is achieved by means of solar cells that generate high photovoltages. In a hybrid technology the semiconducting material is interconnected with catalytic material to enhance the process. Concomitantly, catalytic material is being developed to replace the platinum, a highly precious metal, generally employed for hydrogen formation. Significant progress has been made especially for artificial water-splitting by the discovery of a cobalt-based catalyst that can be incorporated into energy conversion devices. In the past few years fully integrated devices operating on these principles have been reported (Reece et al., 2011; Zhao et al., 2012; Abdi et al., 2013).

In spite of a promising 10% energy conversion efficiency—the minimal standard to make an impact at an industrial scale—for some of these devices, they are not yet commercially competitive with fossil fuels and in the past two decades DSSC efficiency could not be significantly improved. The search is therefore for “dirt cheap” materials, available at low fabrication costs but available in great abundance. Chemically synthesized supramolecular systems can act as homogeneous devices that simultaneously enable light absorption, electron transfer and catalysis. Promising novel materials are dyads, triads, or more complex supermolecules (Megiatto et al., 2012) but they have not yet been integrated within a photovoltaic device. Other potentially very cheap molecular systems consist of photovoltaic solar cells made of conducting polymers doped with dye molecules (Liang et al., 2010). Like all molecule-based solar cells, such devices need to address stability issues under long term operation and resilience toward air (Brabec et al., 2010).

Possibly the cheapest material imaginable is the actual photosynthetic machinery found in photosynthetic organisms. At the level of proteins and protein complexes, the catalytic material is ready-made with proven functionality, consists of ubiquitous substances, and is powered by solar energy while assembled by utilizing carbon dioxide. Most research to date focuses on the interconnection of protein complexes with conducting materials. Some fully integrated photovoltaic devices may serve as proof of principle despite very low efficiencies (Das et al., 2004; Mershin et al., 2012). The main target for artificial photosynthesis beyond photovoltaics is the implementation of PS 1 complexes, generating high energy electrons. In conjunction with platinum electrodes or nanoparticles, photogenerated electrons can be employed for hydrogen generation (Grimme et al., 2008; Iwuchukwu et al., 2009). Complemented with PS 2 and a hydrogen evolving enzyme, a complete biohybrid device can be envisioned (Badura et al., 2006). To date only partially integrated systems, consisting of PS1 complexes fused with hydrogenase for homogeneous catalysis (Ihara et al., 2006) or photoelectrochemical catalysis on gold (Krassen et al., 2009) have been reported.

An additional development is the capture of carbon dioxide and its photoelectrochemical conversion to carbonhydrogen molecules (Spinner et al., 2011). Ideally this conversion reaction would yield a gas or fluid fuel that might be easier to handle than hydrogen gas. Future devices incorporating such reactions will likely compare to those currently used for bulk chemical synthesis, allowing the use of existing factories, and hence they could rapidly become cost effective.

## Implications of oxygenic photosynthesis for human space exploration

### Agriculture in space

Photoautotrophic organisms such as green plants and alga, and cyanobacteria, are essential to support human life in long-term stationary or interplanetary missions because they scrub the crew's air of carbon dioxide by athmospheric fixation, produce oxygen, adjust humidity or recycle wastewater, and convert organic wastes back into edible mass. Physical factors in space that might affect photosynthesis and carbon utilization are solar and cosmic radiation, gravity, temperature, hypobaria, humidity, light, and the absence of an Earth magnetic field (i.e., at the Moon and at Mars). Because earth organisms are evolutionary ill prepared for microgravity (10^−6^–10^−3^ g), fractional gravity (0.17 g for the Moon, 0.38 g for Mars), or cosmic radiation, much space research on photoautotrophs has focused on the biochemical or physiological effects of real or simulated low gravity and ionizing radiation. For agriculture in space the deployment of microalgae may be particularly rewarding as some are very nutritious and can be directly consumed with little processing. They also respond much faster to environmental changes and hence are easier to investigate. Moreover, microalgae are generally robust organisms with highly efficient photosynthesis and are ideal organisms for life support systems (LSS) as they can grow in panel- or tube-fitted reactors within limited confinements (see further and review by Saei et al., 2013).

#### Microgravity and ionizing radiation: physical constraints of sustained life in space?

Because of the constant presence of a gravity vector on Earth, plants have learned to use this force for many biological functions and mechanisms. Hence, the loss or change of gravity alters the way in which plants sense and respond to environmental cues. For instance, *Arabidopsis* hypocotyls growing in microgravity at the ISS display a novel phototrophic response to red light, which is suppressed at 1-g simulated gravity using an on-board centrifuge and in ground controls (Millar et al., 2010). Likewise, seedlings pre-treated with red light experienced a more pronounced blue-light-induced phototropism in microgravity as compared to 1-g controls. Later studies at the ISS confirmed these effects and showed that 0.1–0.3 g sufficed to attenuate the red-light-based phototropism while at a gravity of 0.3 g or above, blue-light phototropism was no longer enhanced. Clearly, conditions at the ISS provide a unique environment to address fundamental questions in space biology (Olsson-Francis and Cockell, 2010; Paul et al., 2013). Importantly, plants grown in the reduced gravities of the Moon (0.17 g) and Mars (0.38 g) are not necessarily exposed to gravity-related stress and so might function normally as if they were on 1-g Earth (Kiss, 2014). Still, more studies with more plants are needed to define the exact threshold for gravity as a prerequisite to (normal) plant life.

In terms of radiation, total dose rates^1^ within Low Earth Orbit spacecraft (somewhere between 160 and 2000 km above the Earth's surface) average from 150 μGy d^−1^ to 500 μGy d^−1^ (Benton and Benton, 2001; Goossens et al., 2006; Vanhavere et al., 2008) as compared to the Earth background radiation levels of circa 1–3 μGy d^−1^ at sea level. During the Apollo missions, with lunar surface exploration lasting 21–75 h and full passage through the Van Allen belts, but no enhanced solar activity, dose rates measured between 180 and 1270 μGy d^−1^ (Bailey, 1975). Dose rates at the lunar surface were measured between 200 and 360 μGy d^−1^. Comparably, based on ~300 days of observation by the Mars Curiosity Rover science laboratory (MSL), the daily space radiation dose at Mars' surface averaged at 210 μGy d^−1^ which was enhanced by roughly 30% during the Solar Particle Event (SPE) of 11 and 12 April 2013 (Hassler et al., 2014).

The survivability after irradiation has been documented for some organisms in terms of the acute lethal dose (LD_50_ in parentheses): humans (4 Gy), mice (4.5 Gy), chicken (10 Gy), fruit fly (640 Gy), onion (20 Gy), wheat (43 Gy), potato (120 Gy), tomato (150 Gy), amoeba (1 kGy), tartigrades (5 kGy), algae (60 Gy–1.2 kGy)^2^, and bacteria (60 Gy–30 kGy) (see also http://www.unscear.org). A few cyanobacteria (*Anabaena torulosa*, 5 kGy, (Singh et al., 2010); *Croococcidiopsis* sp. 029, 15 kGy, (Billi et al., 2000) and algae [*Coccomyxa actinabiotis*, 20 kGy, (Rivasseau et al., 2013)] have been reported as being highly resistant against radiation although the underlying mechanisms have not yet been studied. While it is reassuring that yearly radiation dose rates in space or on the Moon or on Mars will remain below 1 Gy so that growth of plants and microalgae or cyanobacteria will not be impaired, it is obvious that such dose rates may cause DNA mutations with potential detrimental effects. Hence, extensive research on the effects of cosmic radiation on these organisms, in particular for chronic exposures, is fully warranted.

#### A brief history on space biology of plants (and microalgae/cyanobacteria)

Plant research in space started in 1971 with a tiny greenhouse called Oasis, at Salyut 1. After many setbacks in the following decade, arabidopsis grown on Salyut 7 finally produced viable seeds and wheat and mustard were later successfully grown (Ivanova et al., 1993; Mashinsky et al., 1994; Salisbury and Clark, 1995). Meanwhile, at the Spacelab module, a European-American venture, unaware of the earlier Soviet results, encountered (and overcame in 1996) the same developmental problems of space-grown plants (Freeman, 1998; Ivanova et al., 1998). The main culprit appeared to be ethylene, which acts as a hormone and is produced by most plant organs (Chaves and Mello-Farias, 2006). On Earth, ethylene is dispersed by air movement but not so in microgravity where it surrounds the plant, enhancing withering and promoting male sterility. Hence, space greenhouses were fitted with fans and ethylene filters and equipped with humidity, CO_2_, temperature, and oxygen sensors so that the seed-to-seed cycle in space could be finally realized (reviewed by Casado, 2006).

Plants have also been studied when exposed to the open space environment in the EXPOSE-E missions (Rabbow et al., 2012) outside the ISS. At low shielding, an average dose rate of 400 μGy d^−1^ was measured, with a total exposure dose of 215 mGy at the lowest shielding (Berger et al., 2012). Remarkably, seeds of *A. thaliana* and *Nicotiana tabacum* (tobacco) could still germinate after 1.5 years exposure—which also included solar UV, cosmic radiation, temperature fluctuations, and space vacuum (Tepfer et al., 2012). During the same EXPOSE-E mission, it was shown that some cyanobacteria and green algae survived the same length of direct exposure (Cockell et al., 2011; Onofri et al., 2012).

#### Technological advances for “green” research in space

Novel lighting technologies for space green houses make use of light-emitting-diodes (LEDs) which are more durable and reliable than the conventional light sources and allow to simulate parts of the spectrum not present in traditional lighting. They can also emit photons within very narrow bands of the spectrum, i.e., only wavelengths needed by the photosynthetic organisms, hence saving energy. The growth of plants (or microalgae/cyanobacteria) on narrow bands of the spectrum is very useful because some chlorophylls use mostly blue and red wavelengths. For instance, red LEDs were used as a photon source in the Astroculture3 growth chamber (Massa et al., 2006).

Advanced methods of soil-free plant growth are crucial to the future of agriculture in space. Such hydroponic and aeroponic systems (http://en.wikipedia.org/wiki/Hydroponics) require less water, allow recycling and better uptake of nutrients, can be better controlled, and can be stacked in confined spaces (“vertical farming”). Although they are energy-demanding as they incorporate lighting, pumping, and air moderation, they may form the key for the long-term colonization of Mars and the moon where nutritious perlite-like minerals could be extracted from local soils and where energy production from solar or nuclear energy is not an important issue. In addition, hydroponic and aeroponic methods can be integrated into bioregenerative life support systems (Paradiso et al., 2014)—see further.

Another important aspect is improved greenhouse equipment. For instance, the Commercial Plant Biotechnology Facility (CPBF; www.nasa.gov) integrates Astroculture™ technologies, state-of-the-art control software, fault tolerance and recovery technologies, and telescience capability. It also includes LED technology (red and blue at 670 and 450 nm, respectively) and a Fluorescent Light Module (FLM) using high output bi-axial tubes to ensure maximum photon flux. Lately, attention is being directed toward plant growth under fractional gravity in a prelude toward space biology in reduced gravities of the Moon and Mars. In 2015, NASA will attempt to grow arabidopsis, basil, sunflowers, and turnips on the Moon (0.17 g gravity) in small aluminum cylinders brought to the Moon by a robotic spacecraft, the Moon Express lander. Later, similar small greenhouses will be tested at Mars.

Today, “-omics” developments have proliferated the amount of genomic information to a staggering level. Since the *Arabidopsis Genome Initiative* in 2000, many more plant genome sequences were obtained, including for instance those of C4 crops sorghum, maize, and millet, and the C3 crops wheat, barley, rice, soybean, flax, mustard, cucumber, tomato, potato, banana, peach, pear, apple, orange, and wild strawberry. The genome sequencing of microbes gained equal pace with currently around 2500 bacterial and 150 eukaryal genomes fully sequenced, including 71 cyanobacteria and 7 green algae (http://genomesonline.org/). More cyanobacterial and algal genome projects are planned or on-going. For plants, genome projects exist for peanut, chestnut, lettuce, sugar beet, water melon, and coffee. This sequence information can be put to use for the genetic modification of space-dedicated crops or biosphere-supporting bacteria, while genetic responses induced by space conditions will be better understood.

### Life-regenerative supporting systems

A person in space needs at least 22.4 kg of food, water, and oxygen per day (Nelson et al., 2003). For a crew of three astronauts flying to Mars and back, with the fastest return currently achievable i.e., without landing on the Martian surface, an estimated 34 tonnes of supplies would be required. It would be impossible to launch and carry this bulk of weight from Earth. Hence material recycling is the only solution and successful food production in space is thus essential. Microalgae, next to plants, claim a prominent role in food supply routes of Moon or Mars stations but they particularly come into play in spacecraft where weight and volume are critical issues (Hendrickx and Mergeay, 2007; Tikhomirov et al., 2007). The green alga *Chlorella* and the multicellular cyanobacterium *Arthrospira* (aka Spirulina) already have proven their nutritious value and are now globally commercialized—for full review see Chacón-Lee and González-Mariño (2010).

The purpose of Life Support Systems (LSS) is to transform waste and regenerate air, water, and food. Whereas physico-chemical LSS are based on filtration rounds, chemical treatments, and transition processes, bioregenerative LSS (BLSS) use microorganisms and/or plants for oxygen production and carbon dioxide reduction. The main experimental BLSS that use plants and microalgae and that recycle products in a closed loop are summarized in Table 1.

**Table 1:** **The main experimental BLSS that use plants and microalgae and that recycle products in a closed loop**.

**System**	**Country**	**Period**	**Microorganism**	**References**
BIOS	Russia	1960	*Chlorella*	Gitelson et al., 1989
CEBAS^a^	Germany	1985	*Chlamydomonas*	Blüm, 1992; Blüm et al., 2003
MELiSSA^b^	Bel, Fra, Spa, Can	1988	*Arthrospira*	Mergeay et al., 1988; Hendrickx et al., 2006; Lasseur et al., 2011
CERAS^a^	Japan	1997	*Euglena, Chlorella, Arthrospira*	Takeuchi, 1997; Omori et al., 2001; Takeuchi and Endo, 2004
CAES^a^	China	2004	*Chlorella*	Wang et al., 2007

a *contains also an aquatic animal habitat*.

b http://ecls.esa.int/ecls/

**Figure 3:**
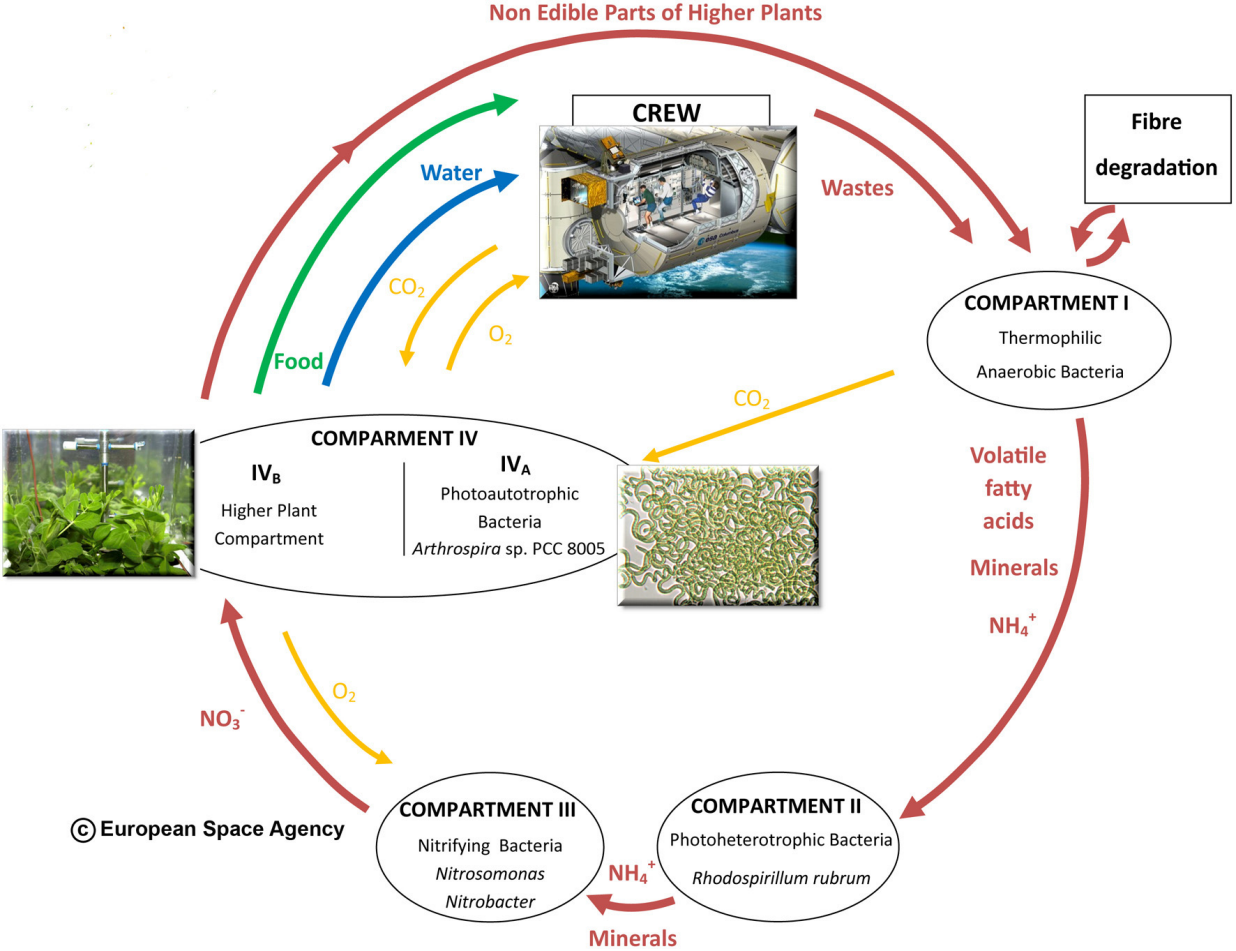
**Scheme presenting the four MELiSSA compartments for the recycling of organic waste into food, water, and oxygen**.

Perhaps the currently best studied system is MELiSSA (Micro Ecological Life Support System Alternative) funded by the European Space Agency (ESA). It consists of four interconnected biological compartments and is completely reliant on photosynthesis. Food and oxygen are produced in the photosynthetic compartment consisting of a plant chamber (IVa) and a microalgae cultivation unit (IVb) (Figure 3). Preferred plant species are those that provide protein-rich food with high nutritious value, such as wheat, soybean, and potato, next to other crops like rice, tomato, lettuce, spinach, and onion (Paradiso et al., 2014). For IVb the cyanobacterium *Arthrospira* sp. PCC 8005 was chosen as *Arthrospira* species are efficient photoautotrophs that produce high amounts of digestible proteins (50–70% of biomass), beta-carotene, omega-6 fatty acids, phycocyanin, important minerals, and antioxidants (Koru, 2012). Marked activities of *Arthrospira* on human health include immunomodulation and anticancer, antiviral, and antibacterial activities while it has positive effects against inflammatory allergy, anemia, cytotoxicity, and radiation sickness (for full review see Ravi et al., 2010 and Sotiroudis and Sotiroudis, 2013).

It was recently shown that acute gamma radiation doses of up to 1600 Gy did not affect the photosynthetic capacity of *Arthrospira* as measured by PSII quantum yield and filaments proliferated normally (H. Badri, pers. comm.). This unexpected radiotolerance is reassuring in view of the relatively high radiation dose rates in Space (0.2–1.2 mGy d^−1^). However, the possibility of genetic changes induced in *Arthrospira* by chronic exposure to cosmic radiation cannot be excluded. The photosynthetic *R. rubrum* (which is in principle edible as well although it is not considered a primary food source in the MELiSSA concept) has previously been studied under real and simulated cosmic radiation and microgravity (Mastroleo et al., 2009). Similar work is now planned by the ESA for *Arthrospira* sp. PCC 8005 since its 2010 genome data (Janssen et al., 2010) have been very recently updated in an assembly of six ordered genome segments and a unique tiling microarray for all its genes has been constructed (P. Janssen, unpublished).

Clearly, bioregenerative life support systems such as MELiSSA show much promise, in particular when very high recycling efficiencies close to 100% can be achieved and taking into account that very few moving parts are used so that minimal maintenance is expected. The selection of the most hardy and most useful photosynthesisers, whether natural, purposely bred, or transgenic, is a challenge that can be met (Lehto et al., 2006; Saei et al., 2013). The only possible drawback of biological systems is that they are dynamic and that genetic changes induced by space conditions may lead to diminished functionality of the reactor loop. Hence, important issues for BLSS to be addressed in the near future are strict quality control, early warning mechanisms, guidelines for counter measures, and ultimately the ability of self-repair.

## Conclusions

Space biology underlines the great potential that exists in the exploitation of the photosynthetic system since this addresses various aspects to produce food, including the recycling of materials and waste management for sustainability of life. Also, technological advances in green space research including lighting, hydro- and aeroponic plant growth, vertical farming, and microbial purification of gray water are generally applicable to improve living conditions on earth. However, in order for such novel applications to make an impact on society, proper technology and knowledge transfer must be guaranteed.

In addition, it will be important to address not just the quantitative aspect of production (e.g., higher yields) but also the qualitative side, sustaining a bio-based economy by also growing minor crops in environmental niches, the efficient use of resources, and an adequate input control. In the present era of climatic, economical, and societal changes, a better understanding of such a fundamental process as photosynthesis is expected to bring about important new technological and scientific initiatives.

Ideally, knowledge acquisition should drive sustainable, science-based innovation. In this, scientists must face their responsibility by communicating to the wider public that, for the development of a sustainable life, much can be learned and achieved by the keen observation of natural processes.

### Conflict of interest statement

The authors declare that the research was conducted in the absence of any commercial or financial relationships that could be construed as a potential conflict of interest.

## Footnotes

^1^Dose rates represent the amount of energy deposited per unit mass, with 1 Gy being 1 J/kg. Health risks are generally expressed in mSv/yr, applying organ-specific and radiation type-specific quality factors.

^2^IAEA Technical Report series 172, Vienna, Austria, 1976.
